# Iodine-Rich Nanoadjuvants for CT Imaging–Guided Photodynamic Immunotherapy of Breast Cancer

**DOI:** 10.3389/fbioe.2022.915067

**Published:** 2022-08-17

**Authors:** Xiaoyan Xin, Xiaoyue Ni, Kang Shi, Jie Shao, Yanqiu Zhang, Xin Peng, Wen Yang, Chuanshuai Tian, Wen Zhou, Bing Zhang

**Affiliations:** ^1^ Department of Radiology, Nanjing Drum Tower Hospital, The Affiliated Hospital of Nanjing University Medical School, Nanjing, China; ^2^ Key Laboratory for Organic Electronics and Information Displays, Jiangsu Key Laboratory for Biosensors, Institute of Advanced Materials (IAM), Jiangsu National Synergetic Innovation Center for Advanced Materials (SICAM), Nanjing University of Posts and Telecommunications, Nanjing, China; ^3^ The Comprehensive Cancer Center of Drum Tower Hospital, Medical School of Nanjing University, Clinical Cancer Institute of Nanjing University, Nanjing, China

**Keywords:** nanoadjuvant, immunotherapy, photodynamic therapy, breast cancer, CT imaging

## Abstract

Immunotherapy, which stimulates the body’s own immune system to kill cancer cells, has shown great promise in the field of cancer therapy. However, the uncontrolled biodistribution of immunotherapeutic drugs may cause severe side effects. Herein, we report an iodine-rich nanoadjuvant (INA) for photo-immunotherapy. INA is prepared by encapsulating a toll-like receptor 7 agonist (R837) and a photosensitizer (phthalocyanine) into an iodine-rich amphiphilic copolymer PEG-PHEMA-I. By virtue of the enhanced permeation and retention (EPR) effect, INA can effectively accumulate into the tumor site. Under light irradiation, photodynamic therapy (PDT) triggered by INA will induce immunogenic cell death (ICD) in the tumor region to trigger the release of immune-associated cytokines. Such a process may further induce the maturation of dendritic cells which will be accelerated by R837, leading to the proliferation of effector T cells for immunotherapy. The photo-immunotherapy mediated by INA shows good anticancer efficacy both *in vitro* and *in vivo*. Meanwhile, INA is also a CT contrast agent owing to its high density of iodine, which can successfully illuminate tumors by CT imaging. Thus, our study develops a light-triggered nanoadjuvant for CT imaging–guided enhanced photo-immunotherapy.

## Introduction

Immunotherapy is a kind of promising cancer therapeutic modality that stimulates the body’s own immune system to attack cancer cells (Pardoll, 2012; Intlekofer and Thompson, 2013; Topalian et al., 2015). Until now, several immunotherapeutic modalities have been successfully applied, such as chimeric antigen receptor (CAR) T cell, immune checkpoint blockade, and cytokine therapy (Pardoll, 2012; Intlekofer and Thompson, 2013; Hargadon et al., 2018). However, because of the uncontrolled tissue accumulation of immunotherapeutic drugs, immunotherapy may suffer from the issue of immune-related adverse events (IRAEs), including hypokalemia, hypophysitis, myocarditis, and thyroid dysfunction (Kumar et al., 2017; Pollack et al., 2017; Darnell et al., 2020). To address such an issue, increasing the tumor accumulation of immunotherapeutic agents is a key point (Lu et al., 2018; Liu et al., 2019). Compared with small-molecular anticancer drugs, nanomedicines have been proven to exhibit better tumor accumulation (Shi et al., 2017; Tran et al., 2017; Youn and Bae, 2018; Miao et al., 2019; Wang X. et al., 2021). By virtue of such advantage, numerous nanomaterials have been designed for cancer immunotherapy to achieve a better therapeutic efficacy with lower IRAEs (Meng et al., 2019; Ji et al., 2020; Mai et al., 2020; Allen et al., 2021). In addition, owing to the multifunctionality of nanomaterials, different kinds of immune stimulants and agonists, and therapeutic modalities, can be easily integrated into one system (Meng et al., 2019; Wang H. et al., 2019; Wang T. et al., 2019; Guo et al., 2021; Wang Z. et al., 2021). Therefore, developing novel nanomedicines is of great significance for cancer immunotherapy.

Despite the improved tumor accumulation of nanomedicine, most therapeutic modalities for immunotherapy activation still have the issue of low specificity. Among different modalities, phototherapy which uses light as an exogenous stimulus has the advantages of high spatiotemporal resolution and low side effects (Chen et al., 2016; Jia et al., 2020). Such a feature makes light an ideal choice for immunotherapy as it can ensure the therapeutic process and induction of immunogenic cell death (ICD) only to occur in the tumor site (Xu et al., 2017; Ding et al., 2018; Li et al., 2019). Until now, nanomaterial-based phototherapy-induced ICD has been applied for treating both primary and metastatic tumors *in vivo* (Wang Z. et al., 2019). In addition to ICD, such a nanosystem may also trigger the delivery of inhibitors, enzymes, adjuvants, and hydrogen sulfide to enhance the immunotherapeutic efficacy (Han et al., 2017; Shi et al., 2018; Wang H. et al., 2019; Sun et al., 2021; Zhang et al., 2022). All of these examples indicate the great promise of nanomaterials in light-induced immunotherapy. But how to integrate light-induced nano-immunotherapy with reliable clinical imaging methods *via* a facile strategy is still a challenge. The combination of a reliable clinical imaging method can not only help examine the location of the tumor but also benefit the observation of the curative effect evaluation of light-induced immunotherapy.

**FIGURE 1:**
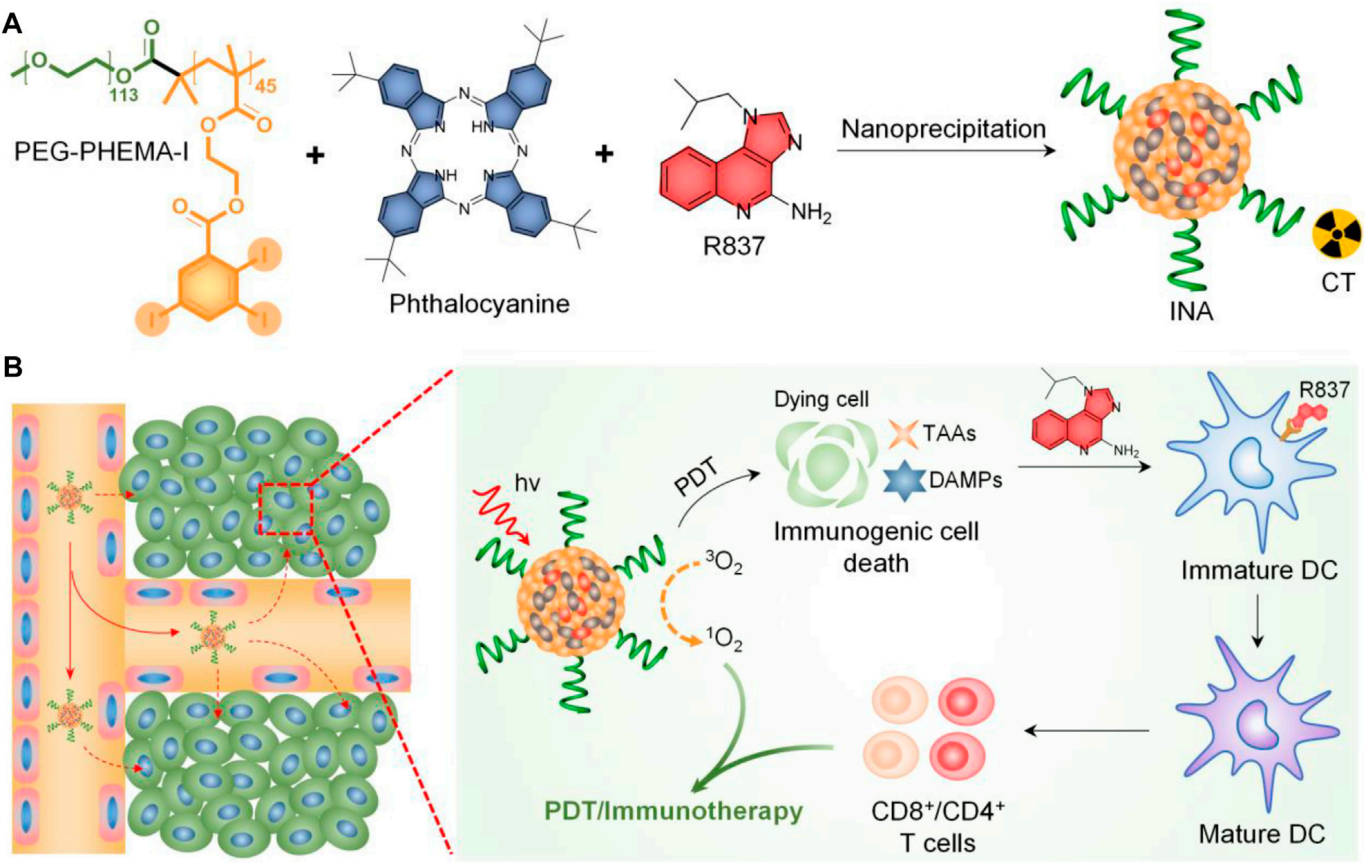
**(A)** Schematic illustration of INA preparation. **(B)** Schematic illustration of the mechanism of INA for NIR light synergistic photodynamic immunotherapy.

Herein, we report iodine-rich nanoadjuvants (INAs) for photodynamic immunotherapy. Such a nanoadjuvant is prepared by co-encapsulating a near-infrared (NIR)–absorbing phthalocyanine and a toll-like receptor-7 (TLR7) agonist (R837) into an iodine-rich amphiphilic copolymer, PEG-PHEMA-I (Figure 1A). After accumulation into the tumor site *via* enhanced permeation and retention (EPR) effect, INA may conduct photodynamic therapy (PDT) under NIR light irradiation. The ICD induced by PDT subsequently triggers the release of danger-associated molecular patterns (DAMPs) and tumor-associated antigens (TAAs) from dying tumor cells. The ICD combined with released R837 will trigger the maturation of dendritic cells (DCs), which further promote the proliferation of effector T cells. As a result, photodynamic immunotherapy is achieved by INA under NIR light irradiation (Figure 1B). In addition, the high concentration of iodine makes INA an ideal candidate for X-ray computed tomography (CT) contrast agents. Thus, INA can be applied as a CT imaging–guided photodynamic immunotherapy for cancer.

## Experimental

### Materials

All the chemicals and solvents are purchased from Sigma-Aldrich and used as received unless otherwise mentioned. PEG-PHEMA-I was synthesized based on our previous study (Zhou et al., 2020).

#### Characterizations

Dynamic light scattering (DLS) was performed on a Brookhaven ZetaPALS. Transmission electron microscopy (TEM) images were captured on an HT7700 transmission electron microscope (acceleration voltage 100 kV). Absorption and fluorescence spectra were recorded on a Shimadzu UV-3600 ultraviolet–visible-near-infrared spectrophotometer and a Fluoromax-3 spectrophotometer (JobinYvon), respectively.

#### Preparation of Nanoparticles

INA was prepared *via* a nanoprecipitation method. Briefly, PEG-PHEMA-I, phthalocyanine, and R837 were dissolved into 1 ml of tetrahydrofuran (THF). The resulting solution was rapidly injected into 9 ml of water under vigorous sonication for 1 min. THF was then removed by gentle nitrogen gas flow, and the nanoparticle solution was filtered through a 0.22-μm filter to remove the aggregates. The obtained solution was stored at 4°C for further usage. The preparation of PEG-PHEMA-I nanoparticles was based on a similar procedure without the addition of R837.

#### Cellular Uptake

Fluorescein isothiocyanate (FITC) (5% w/w) was doped into INA to give INA-FITC a strong green fluorescence signal for confocal imaging. 4T1 cells were cultured in Dulbecco’s modified Eagle’s medium (DMEM) with 10% fetal bovine serum (FBS) and antibiotics (10 mg/ml of streptomycin and 10 U/mL of penicillin) at 37°C in an atmosphere of 5% carbon dioxide. The cells were then seeded into confocal dishes, and INA-FITC (4 μg/ml) was co-incubated with the cells for 4 h. After incubation, the cells were washed with fresh DMEM, and the cell nuclei were stained with DAPI. The cells were then imaged by an LSM880 confocal laser scanning microscope (Carl Zeiss, Germany) with an excitation wavelength of 405 nm for DAPI and 488 nm for INA-FITC.

#### Cytotoxicity Study

4T1 cells were seeded into 96-well plates for 24 h. INA with different concentrations was co-incubated with the cells for another 24 h. The cells were treated with or without 635-nm laser irradiation (1 W/cm^2^) for 10 min. MTT assay was applied to determine the viability of cells.

#### *In Vitro* and *In Vivo* CT Imaging Measurement

Aqueous solutions of INA at different concentrations (3.125 μg/ml, 6.25 μg/ml, 12.5 μg/ml, 25 μg/ml, 50 μg/ml, 100 μg/ml, 200 μg/ml 400 μg/ml, and 400 μg/ml) were directly imaged using Hiscan XM Micro CT (Suzhou Hiscan Information Technology Co., Ltd.). The X-ray attenuation for regions of interest was quantified with Hiscan Analyzer software (Suzhou Hiscan Information Technology Co., Ltd.). 4T1 tumor–bearing mice (*n* = 3) were injected with INA (0.5 mg/ml, 10 μL) and PBS (10 μL) *via* tumor subcutaneous tissue. Then, the mice were scanned using a CT imaging system for 30 min post injection. The 3D reconstruction images and CT values (Hounsfield units, HU) were obtained through RadiAnt DICOM Viewer software.

#### *In Vitro* DC Stimulation Experiments

Bone-marrow-derived dendritic cells were generated from the bone marrow of 8-week-old BALB/c mice from Nanjing Peng Sheng Biological Technology Co. Ltd. according to an established method. For *in vitro* DC stimulation experiments, we cocultured free R837, PEG-PHEMA-I, or INA nanoparticles [(PEG-PHEMA-I) = 100 μg/ml (phthalocyanine) = 25 μg/ml (R837) = 15 μg/ml] with 10^6^ bone-derived DCs from BALB/c mice for 48 h. After various treatments, DCs stained with anti–CD11c-BB700 (BD Biosciences), anti–CD86-PE-Cy7 (BD Biosciences), and anti–CD80-APC (BD Biosciences) antibodies were then analyzed by flow cytometry (CytoFLEX LX). Data analysis was carried out using FCS express software.

#### *In Vito* DC Stimulation Experiments

After 4T1 tumors grown on BALB/c mice reached 100 mm^3^, 200 μL of PEG-PHEMA-I or INA (0.2 mg/ml PEG-PHEMA-I, 0.12 mg/ml R837) was injected intravenously. After injection for 48 h, the mice were exposed to a 680-nm laser with 0.5 W/cm^2^ for 30 min with 1-min interval for every 2 min of light exposure. The mice were killed 2 days after PDT treatment, the draining lymph nodes were cut off, and we assessed the expression levels of CD80 and CD86 by flow cytometry.

#### Cytometric Bead Array Analysis of Cytokines

The cytokines from sera and DC medium supernatants were measured by the BD CBA mouse Th1/Th2 kit according to the manufacturer’s protocol (BD Biosciences) with an appropriate diluent. The samples were run and FACS data were collected using an Accuri C6 Plus (BD Biosciences) flow cytometer and analyzed using FCAP version 3.0 array software (Soft Flow).

#### *In Vivo* PDT

The Ethics Committee of Nanjing Drum Tower Hospital approved all experiments in this study. All animal procedures were carried out in compliance with the guidelines set by the Animal Care Committee at Nanjing Drum Tower Hospital (Nanjing, China). The investigators were not blinded for animal studies. All efforts were made to minimize the number of animals used and their suffering. The mice were randomized on the basis of age and weight.

Female BALB/c mice (6–8 weeks) were purchased from Nanjing Peng Sheng Biological Technology Co. Ltd. and used under protocols approved by Drum Tower Hospital Animal Center. The mice were divided into groups randomly. For tumor inoculation, 4T1 cells (5 × 10^4^) suspended in PBS were subcutaneously injected into the left flank of each female BALB/c mouse. The tumor volume was calculated according to the following formula: width^2^ × length × 0.5. When those tumors reached ∼100 mm^3^, PEG-PHEMA-I or INA nanoparticles were injected into the vein at the INA dose of 0.8 mg per mouse. After 24 h, the mice were irradiated with a 680-nm laser with a power density of 0.5 W/cm^2^ for 30 min with 1 min interval for every 2 min of light exposure.

#### *In Vivo* Immune Response Analysis

To analyze the immune cells in the tumors, those tumors mentioned above were harvested from mice and digested using 1500 U/mL collagenase (Sigma), 1000 U/mL hyaluronidase (Sigma), and Sigma DNase (Sigma) at 37°C for 30 min. The cells were filtered through nylon mesh filters and washed with PBS containing 1% FBS. The single-cell suspension was incubated with anti–CD3-AF700 (BD Biosciences) antibody. Afterward, those cells were washed with PBS containing 1% FBS and analyzed using flow cytometry analysis. Data analysis was carried out using FCS express software.

### Statistical Analysis

The Graphpad Prism 5.0 software (Graphpad Software, La Jolla, CA, United States) was used for all statistical analyses. The mean ± SEM was determined for each treatment group in the individual experiments, and Student’s t-test was used to determine the significance between the treatment and control groups. The results of multiple groups including tumor volume curves have been compared using the variance (ANOVA) test. *p*-values < 0.05 were significant, as indicated with asterisks (∗*p* < 0.05, ∗∗*p* < 0.01, ∗∗∗*p* < 0.001, and ∗∗∗∗*p* < 0.0001).

## Results and Discussion

**FIGURE 2:**
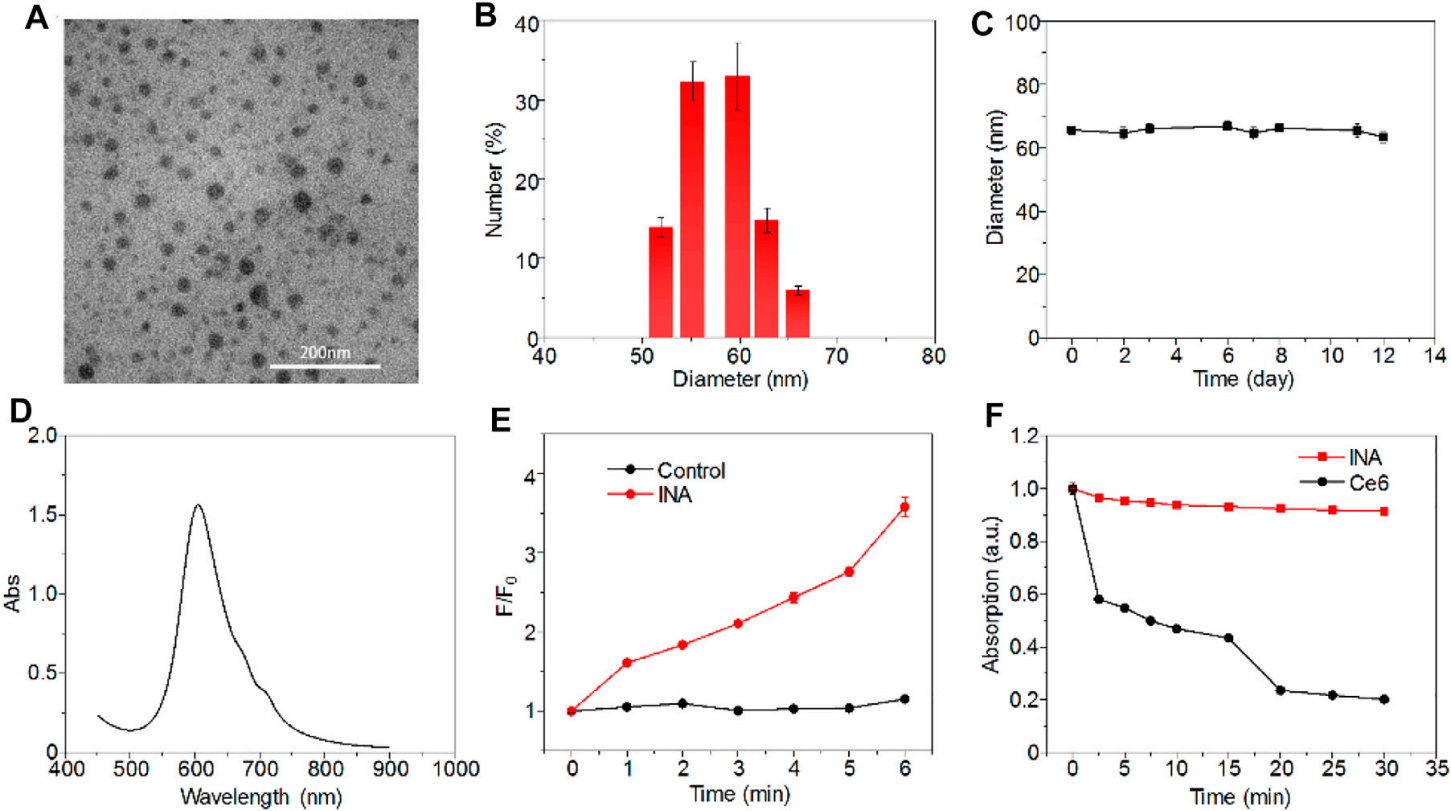
Characterizations of INA. **(A)** Representative TEM image of INA. **(B)** Hydrodynamic size distribution of INA. **(C)** Average hydrodynamic size of INA is a function of storage time. **(D)** Absorption spectrum of INA. **(E)** Fluorescence intensity change (F/F_0_) of SOSG with or without INA as a function of 635-nm laser irradiation time. **(F)** Normalized absorption changes of INA and phthalocyanine at 660 nm as a function of 635-nm laser irradiation time (0.1 W cm^−2^). Error bars represent the standard deviations of three different measurements.

INA was prepared *via* a nanoprecipitation method, which used iodine-grafted amphiphilic copolymer PEG-PHEMA-I as the carrier to load hydrophobic phthalocyanine and R837. The color of the INA solution was blue, and the solution was transparent without the precipitate (Supplementary Figure S1). Transmission electron microscopy (TEM) imaging showed that INA had a spherical morphology with a diameter of approximately 30 nm (Figure 2A). The XRD spectrum of INA was recorded (Supplementary Figure S2). The hydrodynamic size of INA was calculated as 56 nm from dynamic light scattering (DLS), which was much higher than the size measured from the TEM image (Figure 2B). Such difference can be attributed to the shrinkage of nanoparticles during TEM sample preparation. The physiological stability of INA was tested by storing nanoparticle samples under 4°C, and DLS was used to determine the average hydrodynamic size of INA over time. The size remained almost the same even after storing for 12 days, indicating the good stability of INA (Figure 2C). The optical properties of INA were then studied. INA showed maximum absorption at around 600 nm, which was derived from the absorption of encapsulated phthalocyanine (Figure 2D). Almost no fluorescence was detected for INA maybe because of the self-quenching of phthalocyanine in the core of INA. A singlet oxygen (^1^O_2_) fluorescence probe and a singlet oxygen sensor green (SOSG) were then used to study the ^1^O_2_ generation of INA. Although INA showed no fluorescence emission, obvious fluorescence enhancement was observed for SOSG treated with INA under 635-nm laser irradiation, indicating the generation of ^1^O_2_ from INA (Figure 2E). Such a phenomenon may be attributed to the high concentration of iodine within INA, which can enhance the ^1^O2 quantum yield according to our previous study. Under laser irradiation for 30 min, the absorption of INA was nearly unchanged, while the absorption of phthalocyanine decreased 80% under the same condition (Figure 2F). Such a result demonstrated the superior photostability of INA. These data indicated that INA had good physiological and photo-stability and was a good candidate for PDT.

**FIGURE 3:**
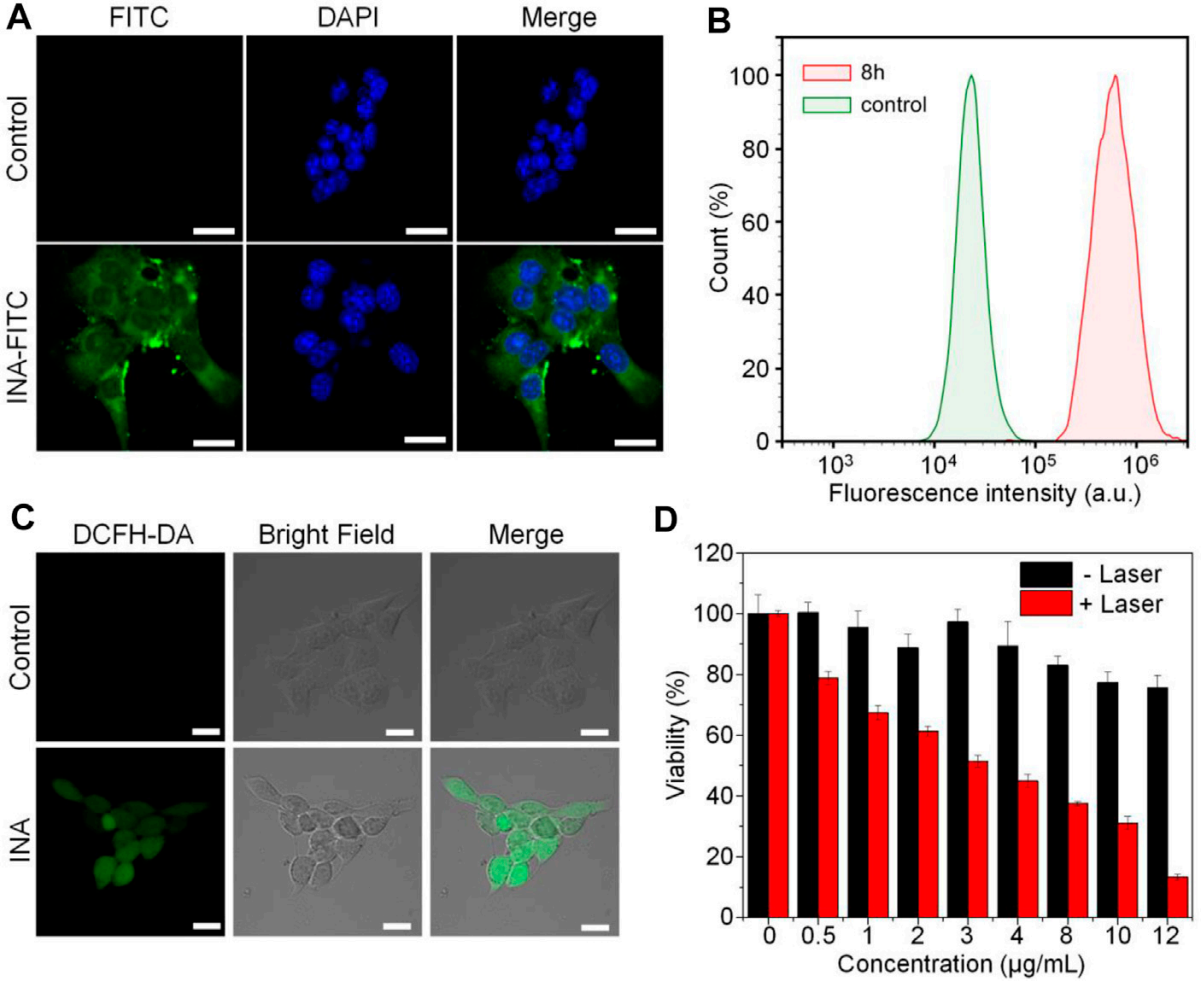
Cell studies. **(A)** Confocal images of 4T1 cells with or without INA-FITC incubation for 8 h. The cell nuclei were stained with DAPI which indicated as blue color. **(B)** Quantification of fluorescence intensity in 4T1 cells incubated with INA-FITC for 8 h by flow cytometry. **(C)** Confocal images of 4T1 cells incubated with INA and DCFH-DA under 635-nm laser irradiation for 12 min **(D)** Cell viability of 4T1 cells after incubating with INA for 24 h at different concentrations with or without 635-nm laser irradiation (1 W cm^−2^). The scale bars represent 20 μm. The error bars represent the standard deviation of three separate measurements.

INA was then applied for cell studies to evaluate its *in vitro* biocompatibility and anticancer efficacy, 4T1 cell was chosen as the cell model. As INA showed no fluorescence signal, 5% w/w fluorescein isothiocyanate (FITC) was loaded into INA to give INA-FITC. INA-FITC showed a strong green fluorescence signal, which was suitable for confocal imaging. After incubating INA-FITC with 4T1 cells for 8 h, strong green fluorescence was detected in the cytoplasm of cells, which showed that INA-FITC can be effectively internalized into 4T1 cells (Figure 3A). A quantification study showed that INA-FITC-incubated cells had a much higher fluorescence intensity than the cells without INA-FITC, further confirming the successful internalization of INA-FITC (Figure 3B). The fluorescence intensity within cells after 8 h incubation was 27-fold higher than that before incubation. To confirm whether INA could generate ^1^O_2_ within 4T1 cells under laser irradiation, a ^1^O_2_ fluorescence probe, dichloro-dihydro-fluorescein diacetate (DCFH-DA), was used. After irradiating 4T1 cells which were incubated with both INA and DCFH-DA by the 635-nm laser for 12 min, a strong green fluorescence signal was detected within cells, while almost no fluorescence signal was detected for the cells without INA incubation (Figure 3C). Such a phenomenon indicated that INA can efficiently generate ^1^O_2_ within 4T1 cells under laser irradiation. Then, *in vitro* biocompatibility and PDT efficacy of INA against 4T1 cells were evaluated by 3-(4,5-dimethylthiazol-2-yl)-2,5-diphenyltetrazolium bromide (MTT) assay. For the cells without laser irradiation, the cell viability was almost 80% even under the highest concentration of INA, indicating the good biocompatibility of INA. In contrast, under laser irradiation, the cell viability decreased gradually with the increase of INA concentration, and the cell viability was only 13.3% at the highest concentration, demonstrating the good *in vitro* PDT efficacy of INA.

Live/dead assay was then utilized to further confirm the PDT of INA against 4T1 cells. 4T1 cells were treated under different conditions, and calcium green (Calcium-AM) and propidium iodide (PI) were stained for live and dead cells, respectively. For the control groups with or without laser irradiation and the INA group without laser irradiation, almost all the cells were alive. However, for the INA group with 635-nm laser irradiation, almost no living cells were observed, which further confirmed the *in vitro* PDT efficiency of INA (Supplementary Figure S3A). The flow cytometry analysis gave similar results, and only both treated with INA and 635-nm laser irradiation can significantly kill 4T1 cells (Supplementary Figure S3B). All these results demonstrated the superior PDT efficacy of INA, which showed its potential for *in vivo* anticancer evaluation.

**FIGURE 4:**
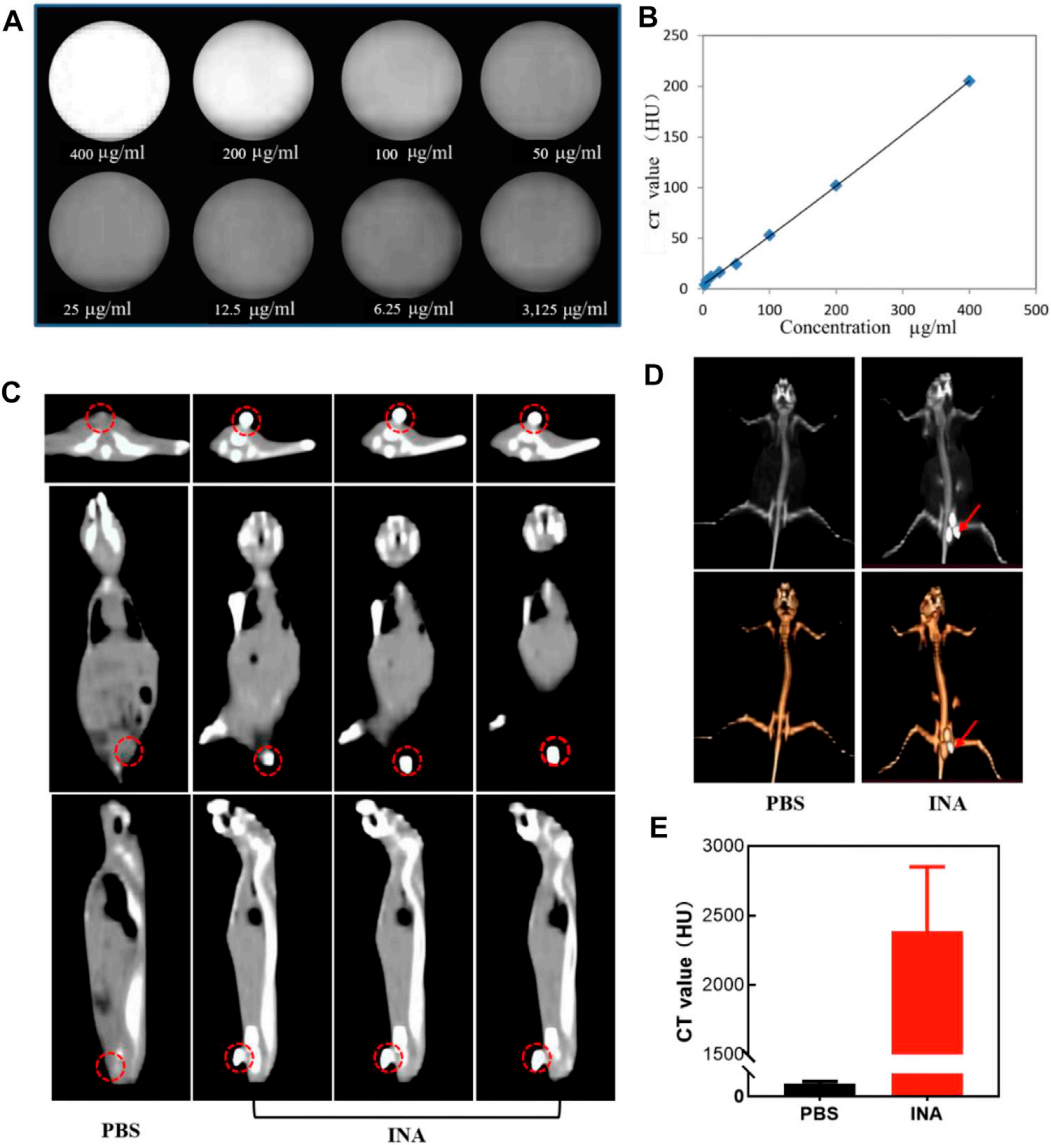
**(A,B)** CT images and the corresponding plot of the CT value (HU) of INA at different concentrations. **(C)** Axial, coronal, and sagittal CT images of 4T1 tumor–bearing mice after intratumoral injection of PBS or INA. Red circles indicate the location of the tumors. **(D)** 3D reconstruction images from CT scan images of 4T1 tumor–bearing mice after intratumoral injection of PBS or INA. Red arrows indicate the location of the tumors. **(E)** Corresponding CT values of tumors after intratumoral injection of PBS or INA.

CT is one of the most extensive imaging techniques for clinical diagnosis due to its deep penetration and high spatial resolution (Schlemmer et al., 2018; Ward et al., 2020; Hricak et al., 2021). The contrast agents used in clinical CT enhancement examination are generally water-soluble compounds containing iodine, which show strong X-ray attenuation capability (Chalela and Aguilar, 2016; Yeh et al., 2017; Zou et al., 2017). Thus, the high density of iodine in INA made it an excellent candidate for positive contrast agents of CT imaging. The CT contrast efficiency of INA was then evaluated, the image brightness was elevated, and the CT value reached about 200 HU at a high concentration (400 μg/ml) of INA (Figures 4A,B). A linear relationship of CT value versus concentration was confirmed (Figure 4B), validating the feasibility of the quantitative study. The ability of *in vivo* CT imaging of INA was further investigated by intratumoral injection of the nanoparticles into 4T1 tumor–bearing mice. The PBS injection group was used as a control. The three pictures on a continuous slice of axial, coronal, and sagittal CT images showed that the tumor which was injected with INA exhibited strong enhancement, while the PBS group showed no obvious contrast (Figure 4C). To observe the enhancement of the tumor site more intuitively, we reconstruct the 3D VR (Virtual Reality) images from the 2D CT images. Severe enhancement of the tumor was detected on the subcutaneous flank of the mice (Figure 4D). The CT values were further analyzed to evaluate the CT imaging ability of INA. The results showed that the CT value of the tumors at 30 min post injection increased over 2000 HU, about forty-fold higher than that of the control group (Figure 4E). These results taken together indicate a strong CT contrast efficiency *in vitro* and *vivo* was achieved by INA.

**FIGURE 5:**
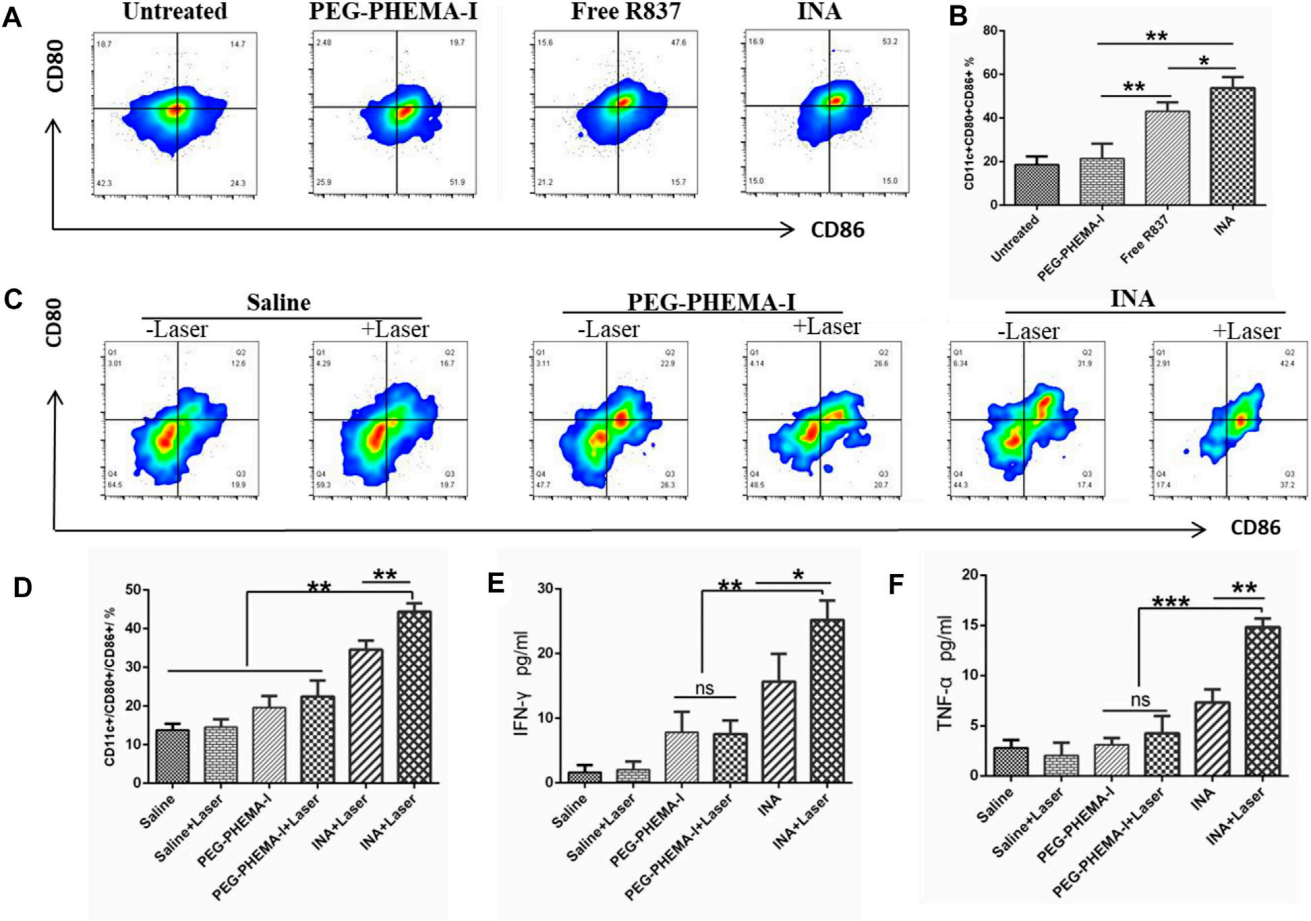
**(A)** Flow cytometry of CD86 and CD80 expressions of DCs after incubation with PEG-PHEMA-I, free R837, or INA for 24 h **(B)** Quantification results of flow cytometry from figure **(A)**. **(C)** Flow cytometry of CD11c, CD80, and CD86 expression of DCs under different treatments. **(D)** Quantification results of flow cytometry from figure **(C) (E,F)** Cytokine levels of IFN-γ and TNF-α in sera from mice isolated on day 2 post INA-based PDT. Three mice were measured in each group. Error bars represent the standard deviation (SD) of at least three replicates.

The immune activation ability of the nanoparticles was then evaluated *in vitro*. Dendritic cells (DCs) are the most potent cells for activating and polarizing naive T cells. DCs are specialized immune cells that scan the surrounding tissue for foreign objects or abnormal cells, and they travel to the lymph nodes to activate T cells and immune response when they spot a danger signal. When they present peptide major histocompatibility complex (MHC) to the T cell receptors (TCR) of T cells, the costimulatory molecules (CD80 and CD86) are upregulated. The surface costimulatory molecules of DCs can reflect the degree of DC maturation. Therefore, we assessed the expression levels of CD80 and CD86 in bone marrow–derived DCs of BALB/C mice treated with PEG-PHEMAA-I, INA, or free R837 for 24 h. Flow cytometry analysis showed that the proportion of mature DC (CD11c+CD80^+^CD86^+^) treated by INA nanoparticles was significantly higher than that induced by the same dose of free R837, while PEG-PHEMAA-I nanoparticles without R837 had no obvious immune response to DC (Figure 5A and Supplementary Figure S4). As shown in Figures 5B,C, the INA group contained more CD11c+CD80^+^CD86^+^ DCs than the free R837 group (53.73 ± 2.899 vs. 43.13 ± 2.322, *n* = 3) and PEG-PHEMA-I group (53.73 ± 2.899 vs. 21.43 ± 3.964, *n* = 3). Taken together, these data represented that INA nanoparticles could act as a strong immune nanoadjuvant.

Recent studies have shown that PDT therapy can activate tumor-specific immune responses by generating tumor-associated antigens from tumor cell residues, which may then be processed by APCs such as DCs and presented to T cells (Chen et al., 2016; Han et al., 2017; Li et al., 2019). We thus wonder whether PDT induced by INA, which could also act as an immune adjuvant, would be able to trigger strong immune responses *in vivo*. After 4T1 tumors grown on BALB/c mice reached 100 mm^3^, 200 μL of PEG-PHEMA-I or INA (0.2 mg/ml PEG-PHEMA-I, 0.12 mg/ml R837) was injected intravenously. After injection for 48 h, the mice were exposed to a 680-nm laser with 0.5 W/cm^2^ for 30 min with a 1-min interval for every 2 min of light exposure. The mice were killed 2 days after PDT treatment, the draining lymph nodes were cut off, and the mature level of DC was detected by flow cytometry. It was found that INA-based PDT therapy promoted a much higher level of DC maturation than that observed for PDT therapy with PEG-PHEMA-I in the absence of R837 (44.43 ± 1.247 vs. 22.43 ± 2.396, *n* = 3) and those with INA injection without laser irradiation (34.57 ± 1.34 vs. 19.60 ± 1.706, *n* = 3), as shown in Figure 5D and Supplementary Figure S5.

Meanwhile, the BD CBA mouse Th1/Th2 kit was used to analyze the changes of IFN-γ, TNF-α, and other cytokines in the serum of mice after different treatments. The expression of important cytokines IFN-γ and TNF-α was significantly upregulated after INA-induced PDT treatment (Figures 5E,F), and their levels were higher than those of PEG-PHEMA-I-induced PDT with laser irradiation (25.19 ± 1.752 vs. 7.570 ± 1.192 for IFN-γ, and 14.82 ± 0.4888 vs. 4.247 ± 1.006 for TNF-α), suggesting that INA-induced PDT had the highest capability for cellular immunity activation *in vivo*. These data indicated that R837 can effectively elevate the PDT-triggered immune response.

**FIGURE 6:**
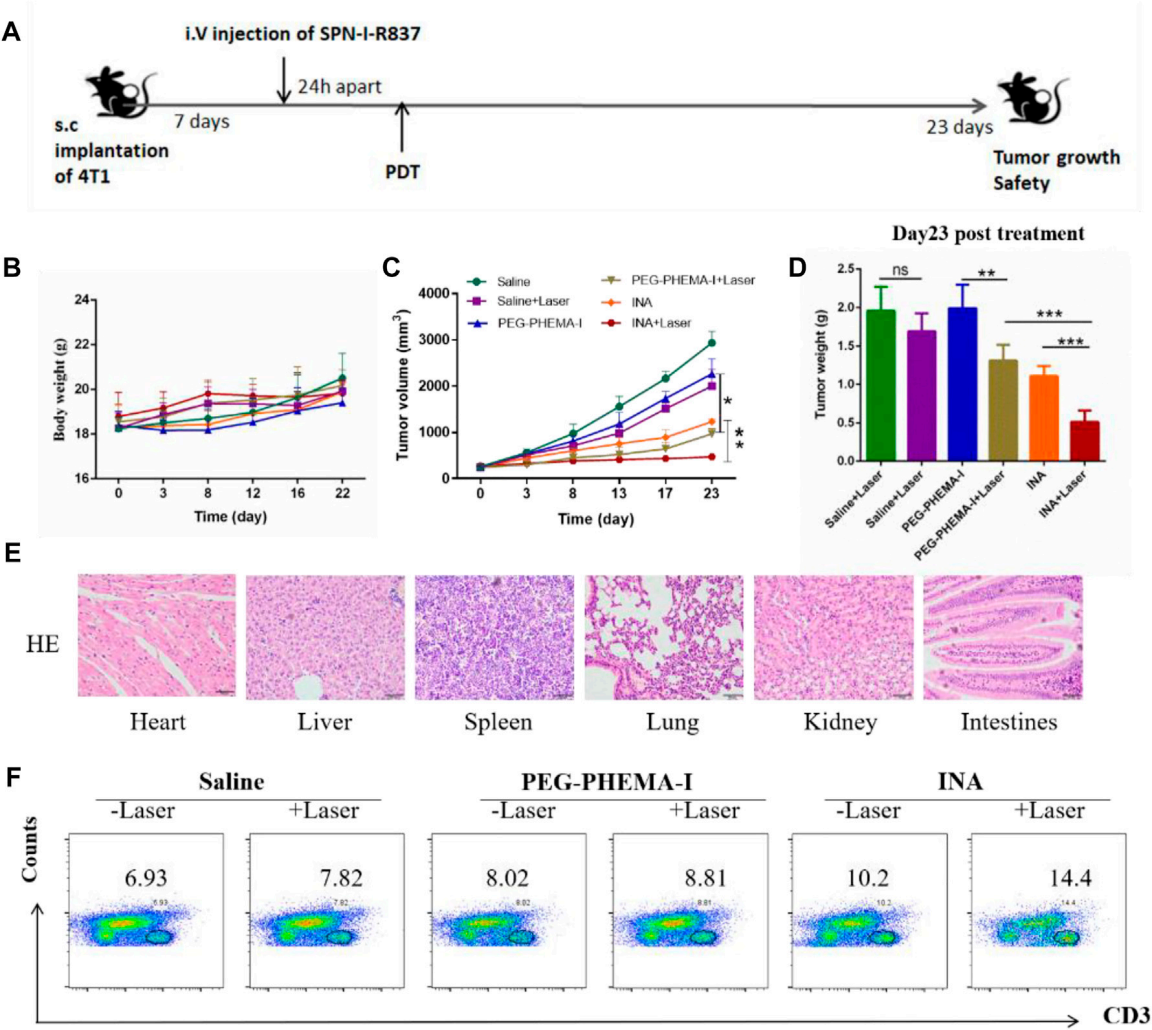
*In vivo* antitumor effects. **(A)** Schematic illustration of our experiment design. Mice with 4T1 tumors were used in our experiment. **(B)** Mice body weight curves of different groups of mice (six mice per group). **(C)** Tumor growth curves of different groups of mice (six mice per group). **(D)** Tumor weight of the treated mice 23 d since the start of the therapy. **(E)** H&E staining of the major organs of INA + laser group of mice. **(F)** Proportions of tumor-infiltrating CD3^+^ T cells.

As INA has been demonstrated to trigger effective immune response under laser irradiation both *in vitro* and *in vivo*, INA was further applied for *in vivo* anticancer study. BALB/c mice bearing subcutaneous 4T1 tumors were intravenously injected (i.v.) with PEG-PHEMA-I or INA and then irradiated by a 680-nm laser at 0.5 W/cm^2^ for 30 min with 1-min interval for every 2 min of light exposure. The design of our animal experiment is shown in Figure 6A. The growth of tumors in different groups was measured by a caliper every 4 days. The photo-immunotherapy triggered by INA had better therapeutic efficacy than PDT-induced by PEG-PHEMA-I nanoparticles, indicating that the presence of R837 can enhance the therapeutic efficacy of photo-immunotherapy (Figures 6C,D and Supplementary Figures S6, S8–S9). The gradual increase of body weight of mice in all the groups demonstrated the safety of treatments (Figure 6B). In addition, no obvious damage was observed from H&E staining of major organs collected from INA-treated mice under laser irradiation, which further proved the biosafety of INA-induced photo-immunotherapy (Figure 6E, Supplementary Figure S7).

The population of CD3^+^ T cells in the tumor region under different treatments was then determined to evaluate the level of immune activation. Flow cytometry results showed that INA-treated mice had a higher population of CD3^+^ T cells in the tumor than PEG-PHEMA-I nanoparticles, demonstrating that R837 can increase the effector T cells in the tumor region. Without laser irradiation, a higher percentage of CD3^+^ T cells was observed in the INA-treated mice (10.2 vs. 8.2). When using laser irradiation, the percentage of CD3^+^ T cells was also higher in the INA-treated mice (14.4 vs. 8.1). The highest population was observed in the INA with the laser group. Such a result indicated that the best anticancer efficacy of INA with laser can be attributed to the highest level of effector T cells in the tumor region (Figure 6F).

## Conclusion

In summary, we have designed a CT imaging–guided photo-immunotherapeutic nanosystem (INA) for cancer theranostics. INA was prepared by co-loading a TLR agonist, R837, and a photosensitizer phthalocyanine into an iodine-grafted amphiphilic copolymer (PEG-PHEMA-I). Under laser irradiation, INA could conduct PDT to trigger ICD, which released immune-related cytokines to induce the maturation of DCs. Meanwhile, R837 in the INA accelerates the maturation of DCs, further leading to the proliferation of effector T cells for immunotherapy. INA showed good ^1^O_2_ quantum yield and photostability. The *in vitro* study indicated that INA could be effectively internalized into cancer cells and killed under NIR laser irradiation. Owing to the high density of iodine, INA had a relatively high CT attenuation coefficient and could indicate the location of the tumor *via* CT imaging. *In vivo* study demonstrated that INA induced the proliferation of cytotoxic T lymphocytes (CTLs) in the tumor region under laser irradiation and could inhibit the tumor growth *via* the photo-immunotherapy.

Our study thus provided a theranostic nanoplatform for imaging-guided photo-immunotherapy. In addition to CT imaging, optical imaging such as fluorescence or PA imaging could be incorporated into the nanoplatform by encapsulating optical materials other than phthalocyanine. In addition, other hydrophobic drugs may be loaded into the nanoplatform to achieve combination therapy for cancer.

## Data Availability

The raw data supporting the conclusions of this article will be made available by the authors, without undue reservation.

## References

[B1] Allen S. D. Liu X. Jiang J. Liao Y.-P. Chang C. H. Nel A. E. (2021). Immune Checkpoint Inhibition in Syngeneic Mouse Cancer Models by a Silicasome Nanocarrier Delivering a GSK3 Inhibitor. Biomaterials 269, 120635. 10.1016/j.biomaterials.2020.120635 33422940 PMC7870571

[B2] Chalela J. G. Aguilar L. (2016). Images in Clinical Medicine. Iododerma from Contrast Material. N. Engl. J. Med. 374 (25), 2477. 10.1056/NEJMicm1512512 27332906

[B3] Chen Q. Xu L. Liang C. Wang C. Peng R. Liu Z. (2016). Photothermal Therapy with Immune-Adjuvant Nanoparticles Together with Checkpoint Blockade for Effective Cancer Immunotherapy. Nat. Commun. 7, 13193. 10.1038/ncomms13193 27767031 PMC5078754

[B4] Darnell E. P. Mooradian M. J. Baruch E. N. Yilmaz M. Reynolds K. L. (2020). Immune-Related Adverse Events (irAEs): Diagnosis, Management, and Clinical Pearls. Curr. Oncol. Rep. 22 (4), 39. 10.1007/s11912-020-0897-9 32200442

[B5] Ding B. Shao S. Yu C. Teng B. Wang M. Cheng Z. (2018). Large‐Pore Mesoporous‐Silica‐Coated Upconversion Nanoparticles as Multifunctional Immunoadjuvants with Ultrahigh Photosensitizer and Antigen Loading Efficiency for Improved Cancer Photodynamic Immunotherapy. Adv. Mat. 30 (52), 1802479. 10.1002/adma.201802479 30387197

[B6] Guo S. Li K. Hu B. Li C. Zhang M. Hussain A. (2021). Membrane‐Destabilizing Ionizable Lipid Empowered Imaging‐guided siRNA Delivery and Cancer Treatment. Exploration 1 (1), 35–49. 10.1002/exp.20210008 PMC10291568 37366466

[B7] Han Q. Wang X. Jia X. Cai S. Liang W. Qin Y. (2017). CpG Loaded MoS2 Nanosheets as Multifunctional Agents for Photothermal Enhanced Cancer Immunotherapy. Nanoscale 9 (18), 5927–5934. 10.1039/c7nr01460k 28436514

[B8] Hargadon K. M. Johnson C. E. Williams C. J. (2018). Immune Checkpoint Blockade Therapy for Cancer: An Overview of FDA-Approved Immune Checkpoint Inhibitors. Int. Immunopharmacol. 62, 29–39. 10.1016/j.intimp.2018.06.001 29990692

[B9] Hricak H. Abdel-Wahab M. Atun R. Lette M. M. Paez D. Brink J. A. (2021). Medical Imaging and Nuclear Medicine: A Lancet Oncology Commission. Lancet Oncol. 22 (4), 136–172. 10.1016/s1470-2045(20)30751-8 PMC8444235 33676609

[B10] Intlekofer A. M. Thompson C. B. (2013). At the Bench: Preclinical Rationale for CTLA-4 and PD-1 Blockade as Cancer Immunotherapy. J. Leukoc. Biol. 94 (1), 25–39. 10.1189/jlb.1212621 23625198 PMC3685017

[B11] Ji Y. Liu X. Li J. Xie X. Huang M. Jiang J. (2020). Use of Ratiometrically Designed Nanocarrier Targeting CDK4/6 and Autophagy Pathways for Effective Pancreatic Cancer Treatment. Nat. Commun. 11 (1), 4249. 10.1038/s41467-020-17996-7 32843618 PMC7447818

[B12] Jia Y. Song Y. Qu Y. Peng J. Shi K. Du D. (2020). Mesoporous PtPd Nanoparticles for Ligand-Mediated and Imaging-Guided Chemo-Photothermal Therapy of Breast Cancer. Nano Res. 13, 1739–1748. 10.1007/s12274-020-2800-2

[B13] Kumar V. Chaudhary N. Garg M. Floudas C. S. Soni P. Chandra A. B. (2017). Current Diagnosis and Management of Immune Related Adverse Events (irAEs) Induced by Immune Checkpoint Inhibitor Therapy. Front. Pharmacol. 8, 49. 10.3389/fphar.2017.00049 28228726 PMC5296331

[B14] Li W. Yang J. Luo L. Jiang M. Qin B. Yin H. (2019). Targeting Photodynamic and Photothermal Therapy to the Endoplasmic Reticulum Enhances Immunogenic Cancer Cell Death. Nat. Commun. 10 (1), 3349. 10.1038/s41467-019-11269-8 31350406 PMC6659660

[B15] Liu X. Zheng J. Sun W. Zhao X. Li Y. Gong N. (2019). Ferrimagnetic Vortex Nanoring-Mediated Mild Magnetic Hyperthermia Imparts Potent Immunological Effect for Treating Cancer Metastasis. ACS Nano 13 (8), 8811–8825. 10.1021/acsnano.9b01979 31328922

[B16] Lu Y. Yang Y. Gu Z. Zhang J. Song H. Xiang G. (2018). Glutathione-Depletion Mesoporous Organosilica Nanoparticles as a Self-Adjuvant and Co-Delivery Platform for Enhanced Cancer Immunotherapy. Biomaterials 175, 82–92. 10.1016/j.biomaterials.2018.05.025 29803106

[B17] Mai X. Zhang Y. Fan H. Song W. Chang Y. Chen B. (2020). Integration of Immunogenic Activation and Immunosuppressive Reversion Using Mitochondrial-Respiration-Inhibited Platelet-Mimicking Nanoparticles. Biomaterials 232, 119699. 10.1016/j.biomaterials.2019.119699 31891817

[B18] Meng Z. Zhou X. Xu J. Han X. Dong Z. Wang H. (2019). Light‐Triggered *In Situ* Gelation to Enable Robust Photodynamic‐Immunotherapy by Repeated Stimulations. Adv. Mat. 31 (24), e1900927. 10.1002/adma.201900927 31012164

[B19] Miao Z. Chen S. Xu C.-Y. Ma Y. Qian H. Xu Y. (2019). PEGylated Rhenium Nanoclusters: A Degradable Metal Photothermal Nanoagent for Cancer Therapy. Chem. Sci. 10 (21), 5435–5443. 10.1039/C9SC00729F 31293725 PMC6544121

[B20] Pardoll D. M. (2012). The Blockade of Immune Checkpoints in Cancer Immunotherapy. Nat. Rev. Cancer 12 (4), 252–264. 10.1038/nrc3239 22437870 PMC4856023

[B21] Pollack M. H. Betof A. Dearden H. Rapazzo K. Valentine I. Brohl A. S. (2017). Safety of Resuming Anti-PD-1 in Patients with Immune-Related Adverse Events (irAEs) during Combined Anti-CTLA-4 and Anti-PD1 in Metastatic Melanoma. Ann. Oncol. 29 (1), 250–255. 10.1093/annonc/mdx642 PMC5834131 29045547

[B22] Schlemmer H.-P. Bittencourt L. K. D’Anastasi M. Domingues R. Khong P.-L. Lockhat Z. (2018). Global Challenges for Cancer Imaging. J. Glob. Oncol. 4, 1–10. 10.1200/jgo.17.00036 PMC6180759 30241164

[B23] Shi B. Yan Q. Tang J. Xin K. Zhang J. Zhu Y. (2018). Hydrogen Sulfide-Activatable Second Near-Infrared Fluorescent Nanoassemblies for Targeted Photothermal Cancer Therapy. Nano Lett. 18 (10), 6411–6416. 10.1021/acs.nanolett.8b02767 30239208

[B24] Shi J. Kantoff P. W. Wooster R. Farokhzad O. C. (2017). Cancer Nanomedicine: Progress, Challenges and Opportunities. Nat. Rev. Cancer 17 (1), 20–37. 10.1038/nrc.2016.108 27834398 PMC5575742

[B25] Sun Y. Feng X. Wan C. Lovell J. F. Jin H. Ding J. (2021). Role of Nanoparticle-Mediated Immunogenic Cell Death in Cancer Immunotherapy. Asian J. Pharm. Sci. 16 (2), 129–132. 10.1016/j.ajps.2020.05.004 33995609 PMC8105413

[B26] Topalian S. L. Drake C. G. Pardoll D. M. (2015). Immune Checkpoint Blockade: A Common Denominator Approach to Cancer Therapy. Cancer Cell 27 (4), 450–461. 10.1016/j.ccell.2015.03.001 25858804 PMC4400238

[B27] Tran S. DeGiovanni P. J. Piel B. Rai P. (2017). Cancer Nanomedicine: A Review of Recent Success in Drug Delivery. Clin. Transl. Med. 6 (1), 44. 10.1186/s40169-017-0175-0 29230567 PMC5725398

[B28] Wang H. Han X. Dong Z. Xu J. Wang J. Liu Z. (2019a). Hyaluronidase with pH‐Responsive Dextran Modification as an Adjuvant Nanomedicine for Enhanced Photodynamic‐Immunotherapy of Cancer. Adv. Funct. Mat. 29 (29), 1902440. 10.1002/adfm.201902440

[B29] Wang T. Zhang H. Han Y. Liu H. Ren F. Zeng J. (2019b). Light-Enhanced O2-Evolving Nanoparticles Boost Photodynamic Therapy to Elicit Antitumor Immunity. ACS Appl. Mat. Interfaces 11 (18), 16367–16379. 10.1021/acsami.9b03541 30994323

[B30] Wang X. Zhong X. Li J. Liu Z. Cheng L. (2021a). Inorganic Nanomaterials with Rapid Clearance for Biomedical Applications. Chem. Soc. Rev. 50 (15), 8669–8742. 10.1039/D0CS00461H 34156040

[B31] Wang Z. Little N. Chen J. Lambesis K. T. Le K. T. Han W. (2021b). Immunogenic Camptothesome Nanovesicles Comprising Sphingomyelin-Derived Camptothecin Bilayers for Safe and Synergistic Cancer Immunochemotherapy. Nat. Nanotechnol. 16 (10), 1130–1140. 10.1038/s41565-021-00950-z 34385682 PMC8855709

[B32] Wang Z. Zhang F. Shao D. Chang Z. Wang L. Hu H. (2019c). Janus Nanobullets Combine Photodynamic Therapy and Magnetic Hyperthermia to Potentiate Synergetic Anti‐Metastatic Immunotherapy. Adv. Sci. 6 (22), 1901690. 10.1002/advs.201901690 PMC6864517 31763151

[B33] Ward Z. J. Scott A. M. Hricak H. Abdel-Wahab M. Paez D. Lette M. M. (2020). Estimating the Impact of Treatment and Imaging Modalities on 5-Year Net Survival of 11 Cancers in 200 Countries: A Simulation-Based Analysis. Lancet Oncol. 21 (8), 1077–1088. 10.1016/S1470-2045(20)30317-X 32758462 PMC8020599

[B34] Xu J. Xu L. Wang C. Yang R. Zhuang Q. Han X. (2017). Near-Infrared-Triggered Photodynamic Therapy with Multitasking Upconversion Nanoparticles in Combination with Checkpoint Blockade for Immunotherapy of Colorectal Cancer. ACS Nano 11 (5), 4463–4474. 10.1021/acsnano.7b00715 28362496

[B35] Yeh B. M. FitzGerald P. F. Edic P. M. Lambert J. W. Colborn R. E. Marino M. E. (2017). Opportunities for New CT Contrast Agents to Maximize the Diagnostic Potential of Emerging Spectral CT Technologies. Adv. Drug Deliv. Rev. 113, 201–222. 10.1016/j.addr.2016.09.001 27620496 PMC5344792

[B36] Youn Y. S. Bae Y. H. (2018). Perspectives on the Past, Present, and Future of Cancer Nanomedicine. Adv. Drug Deliv. Rev. 130, 3–11. 10.1016/j.addr.2018.05.008 29778902

[B37] Zhang S. Wang J. Kong Z. Sun X. He Z. Sun B. (2022). Emerging Photodynamic Nanotherapeutics for Inducing Immunogenic Cell Death and Potentiating Cancer Immunotherapy. Biomaterials 282, 121433. 10.1016/j.biomaterials.2022.121433 35202933

[B38] Zhou W. Chen Y. Zhang Y. Xin X. Li R. Xie C. (2020). Iodine‐Rich Semiconducting Polymer Nanoparticles for CT/Fluorescence Dual‐Modal Imaging‐Guided Enhanced Photodynamic Therapy. Small 16 (5), e1905641. 10.1002/smll.201905641 31898866

[B39] Zou Y. Wei Y. Wang G. Meng F. Gao M. Storm G. (2017). Nanopolymersomes with an Ultrahigh Iodine Content for High-Performance X-Ray Computed Tomography Imaging *In Vivo* . Adv. Mat. 29 (10), 1603997. 10.1002/adma.201603997 28054400

