# Supplementary material for: Iodine-Rich Nanoadjuvants for CT Imaging–Guided Photodynamic Immunotherapy of Breast Cancer
Source: Front Bioeng Biotechnol. 2022 Aug 17;10:915067. doi: 10.3389/fbioe.2022.915067 (PMC9442603; doi:10.3389/fbioe.2022.915067)
Supplement: Supplementary file 1 [file DataSheet1.PDF]

Supporting Information

Iodine-rich Nanoadjuvants for CT Imaging-guided Photodynamic Immunotherapy of Breast Cancer

† Department of Radiology, Nanjing Drum Tower Hospital, The Affiliated Hospital of Nanjing University Medical School, Nanjing, 210008, China.

‡ Key Laboratory for Organic Electronics and Information Displays & Jiangsu Key Laboratory for Biosensors, Institute of Advanced Materials (IAM), Jiangsu National Synergetic Innovation Center for Advanced Materials (SICAM), Nanjing University of Posts & Telecommunications, Nanjing 210023, China.

⊥ The Comprehensive Cancer Center of Drum Tower Hospital, Medical School of Nanjing University & Clinical Cancer Institute of Nanjing University, Nanjing 210008, China.

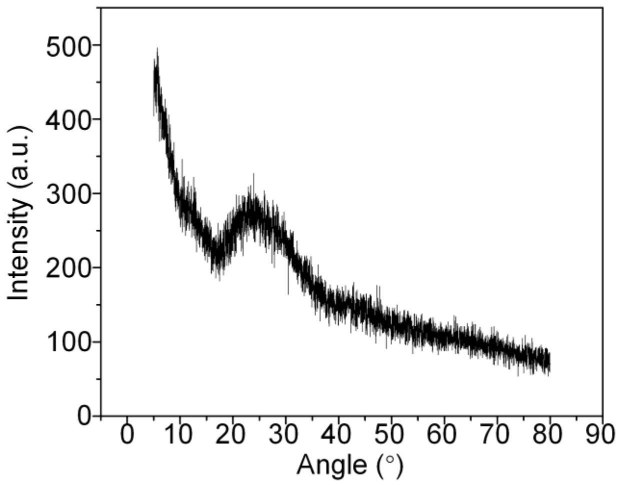

Figure S1. The XRD spectrum of INA

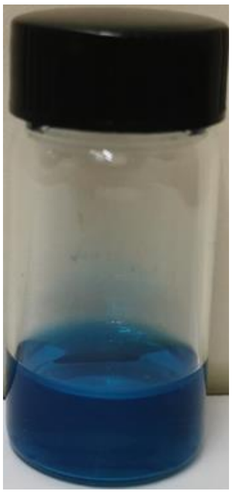

Figure S2. The picture of INA solution

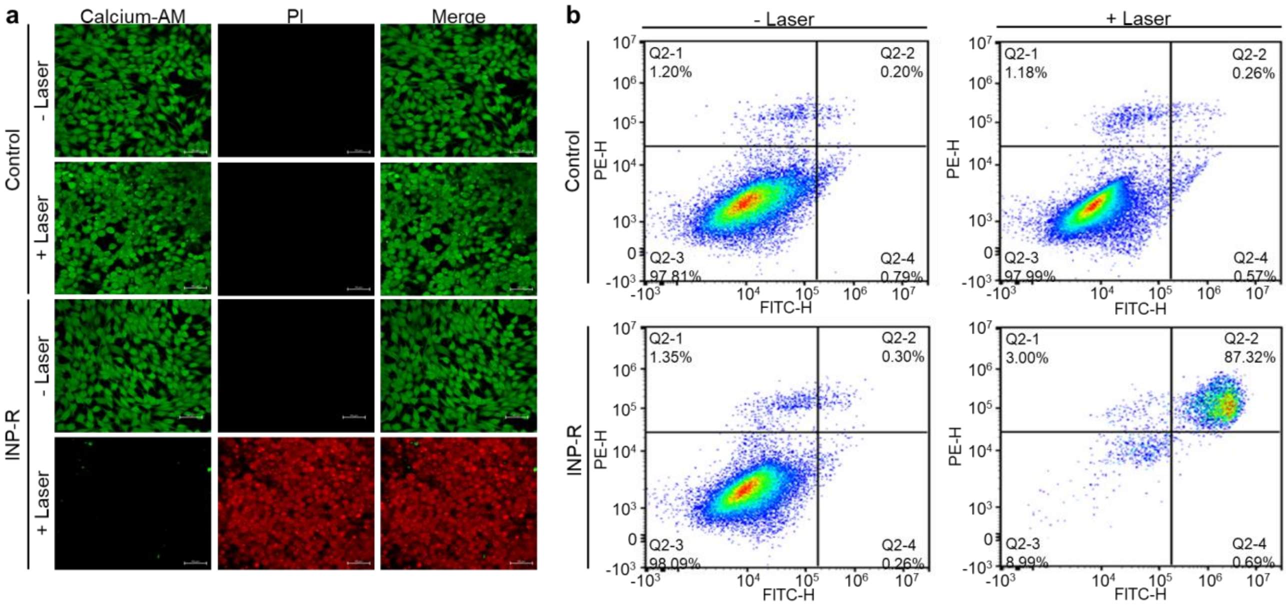

Figure S3. (a) Live/dead assay of 4T1 cells under different treatment with or without 635 nm laser irradiation. (b) Representative flow cytometry plots of 4T1 cell under different treatments.

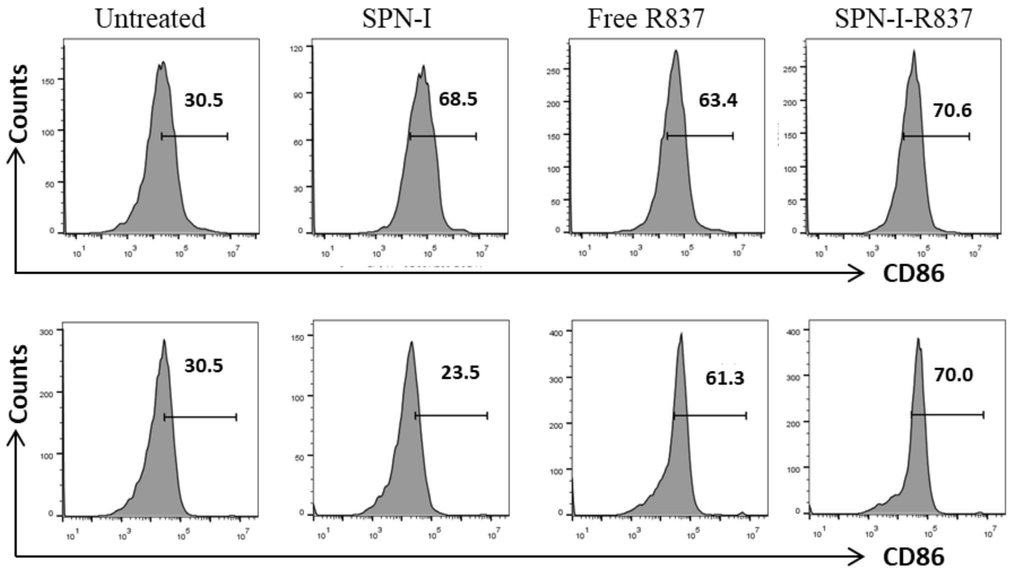

Figure S4. In vitro immune stimulation effect of INA. Flow diagram of CD86 and CD80 expressions, which are markers for DCs maturation, after in vitro incubation of DCs with PEG-PHEMA-I, free R837, or INA for 24h.

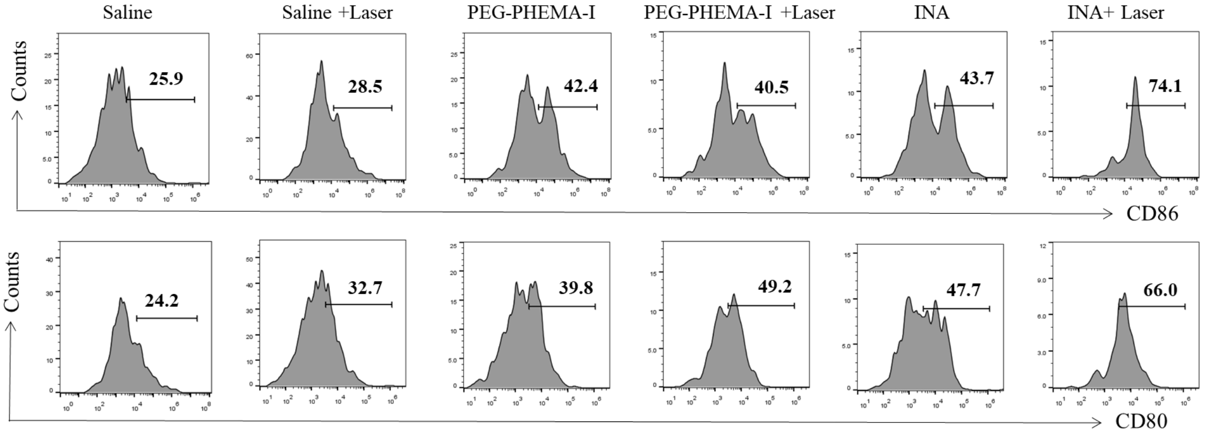

Figure S5. INA-based in vivo PDT induces DC maturation and stimulates the expression of pro-inflammatory cytokines. (a-c) Flow cytometry assessment of collected Cells in the tumor-draining lymph nodes after staining with CD11c, CD80, and CD86.

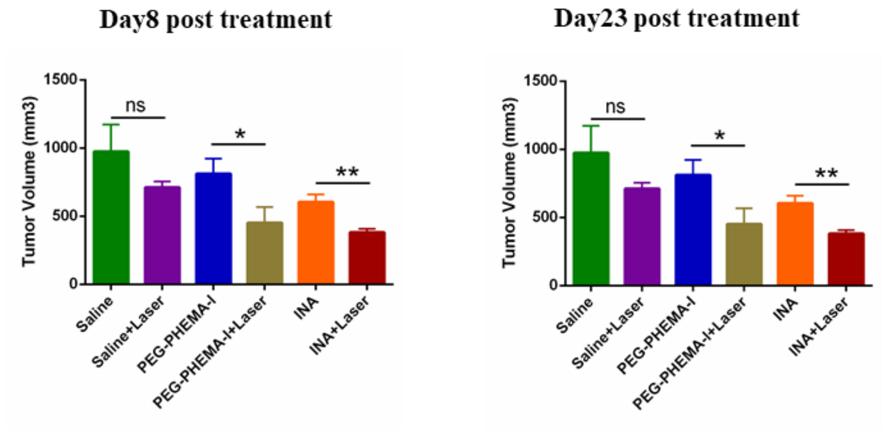

Figure S6. The tumor volume of the treated mice 8 d and 23 d since the start of the therapy.

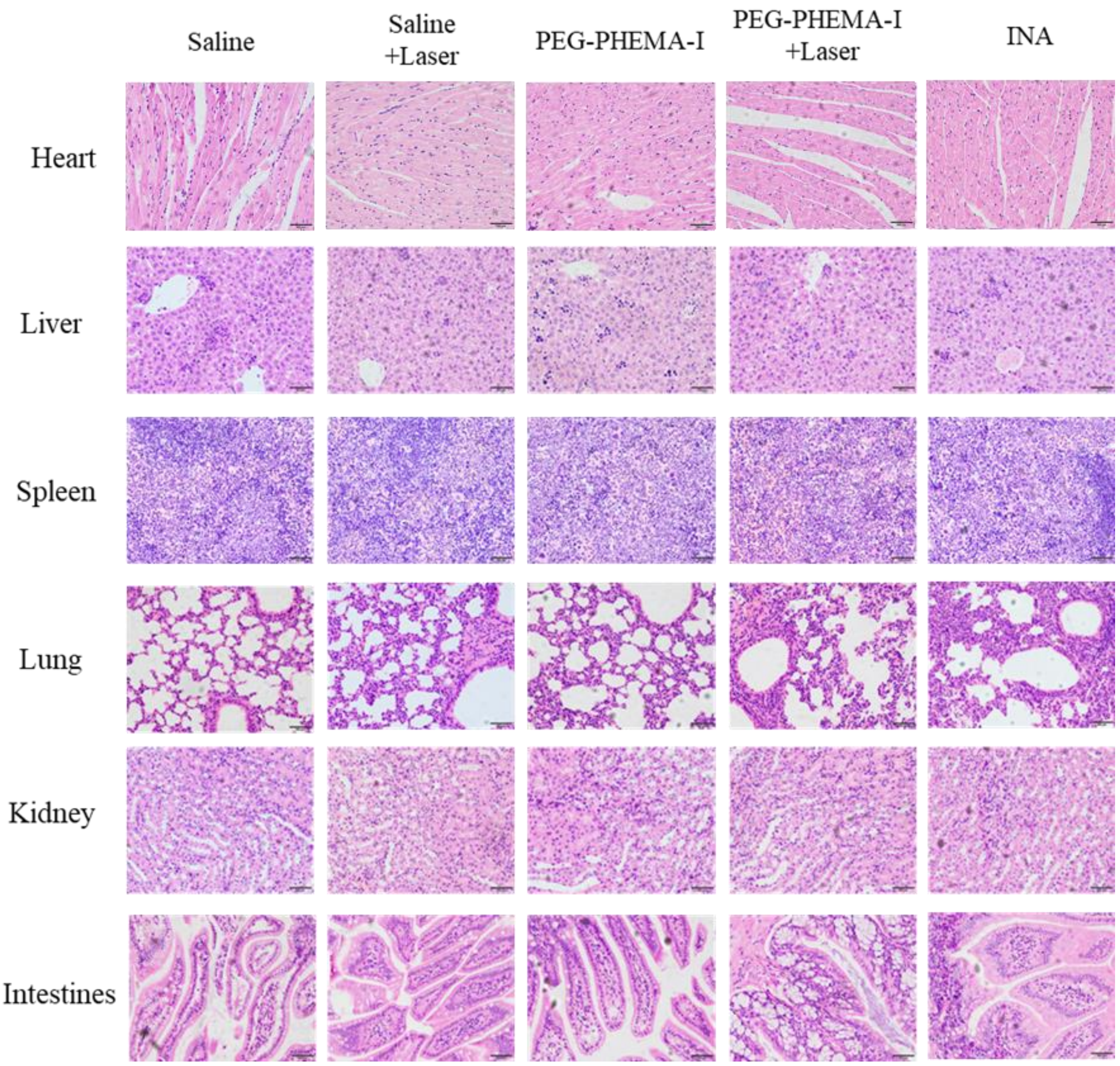

Figure S7. H&E staining of the major organs of saline， saline+laser, PEG-PHEMA-I and PEG-PHEMA-I+laser group.

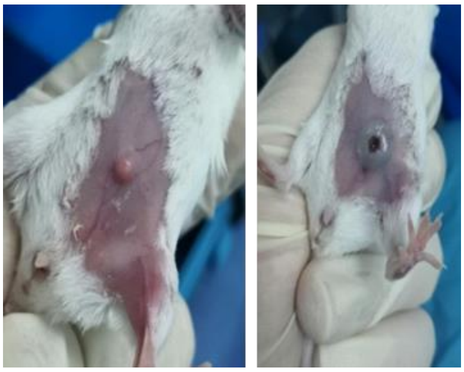

Figure S8 Pictures of mouse tumor-bearing before and after injection of INA+laser.

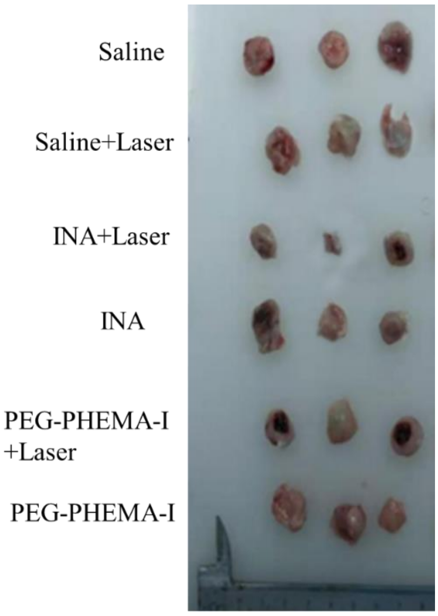

Figure S9 Pictures of tumor issues after various treatments.
